# Supplementary material for: Hedging our bet on forest permanence for the economic viability of climate targets
Source: Nat Commun. 2025 Mar 27;16:2460. doi: 10.1038/s41467-025-57607-x (PMC11950357; doi:10.1038/s41467-025-57607-x)
Supplement: Supplementary file 1 — Supplementary Information [file 41467_2025_57607_MOESM1_ESM.pdf]

# Supplementary Information for Hedging our bet on forest permanence for the economic viability of climate targets

Michael G. Windisch[1,2,3*], Florian Humpenöder[1], Leon Merfort[1], Nico Bauer[1], Gunnar Luderer[1,4], Jan Philipp Dietrich[1], Jens Heinke[1], Christoph Müller[1], Gabriel Abrahao[1], Hermann Lotze-Campen[1,2], and Alexander Popp[1,5,6]

[1]Potsdam Institute for Climate Impact Research - Member of the Leibniz Association; Potsdam, Germany
[2]Humboldt University of Berlin; Berlin, Germany
[3]Institute for Atmospheric and Climate Science, ETH Zurich; Zurich, Switzerland
[4]Global Energy Systems Analysis, Technische Universität Berlin; Berlin, Germany
[5]Kassel Institute for Sustainability; Kassel, Germany
[6]Faculty of Organic Agricultural Sciences, University of Kassel; Witzenhausen, Germany

*Corresponding author. Email: michael.windisch@env.ethz.ch

**The PDF file includes:**

Supplementary Figures 1 to 7
Supplementary Table 1
Supplementary References

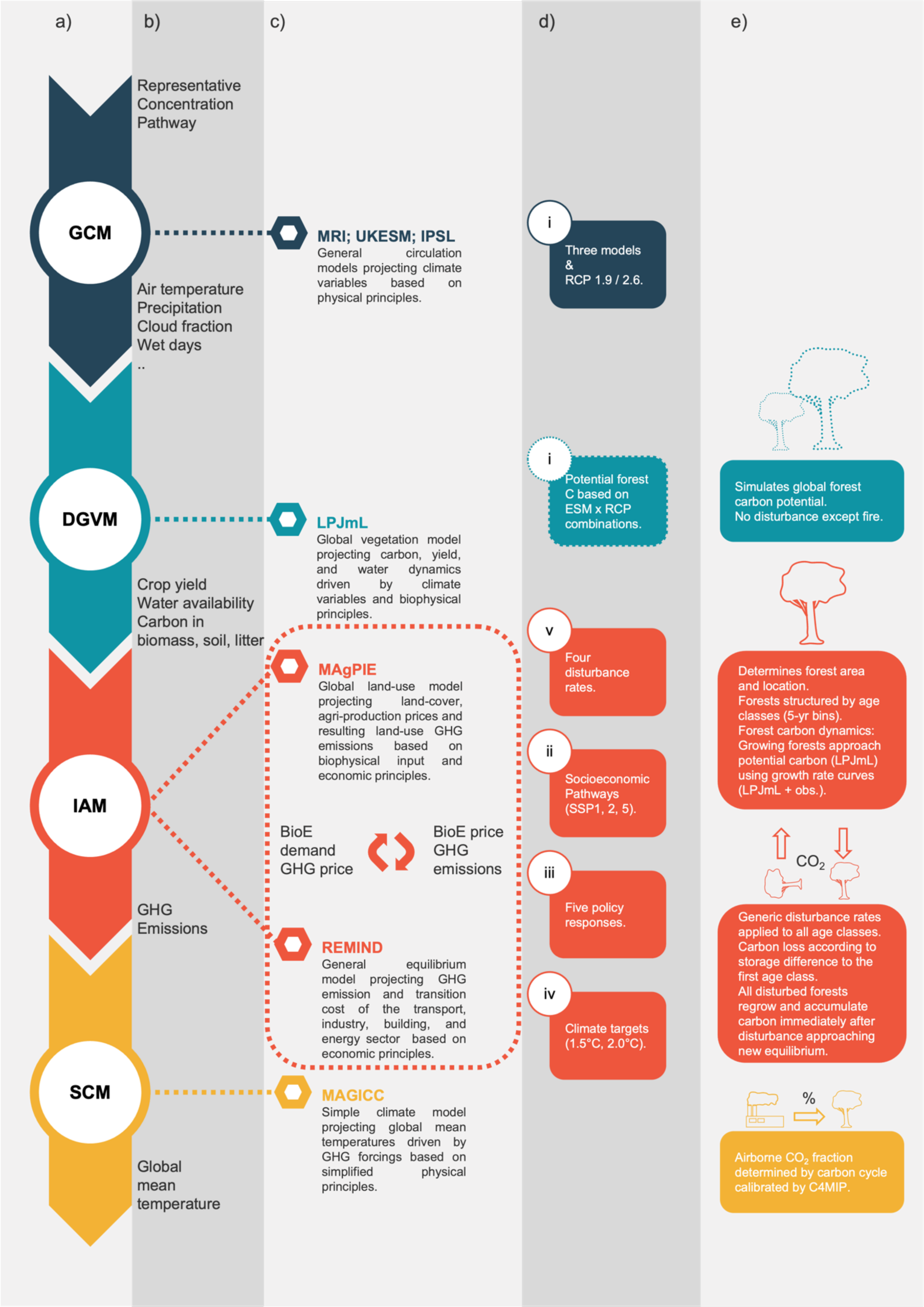

a)
b)
c)
d)
e)
Representative Concentration Pathway
GCM
Air temperature
Precipitation
Cloud fraction
Wet days
..
MRI; UKESM; IPSL
General circulation models projecting climate variables based on physical principles.
i
Three models & RCP 1.9 / 2.6.
DGVM
LPJmL
Global vegetation model projecting carbon, yield, and water dynamics driven by climate variables and biophysical principles.
i
Potential forest C based on ESM x RCP combinations.
Simulates global forest carbon potential.
No disturbance except fire.
Crop yield
Water availability
Carbon in biomass, soil, litter
MAgPIE
Global land-use model projecting land-cover, agri-production prices and resulting land-use GHG emissions based on biophysical input and economic principles.
v
Four disturbance rates.
Determines forest area and location.
Forests structured by age classes (5-yr bins).
Forest carbon dynamics: Growing forests approach potential carbon (LPJmL) using growth rate curves (LPJmL + obs.).
IAM
ii
Socioeconomic Pathways (SSP1, 2, 5).
BioE demand
GHG price
BioE price
GHG emissions
$CO_2$
iii
Five policy responses.
GHG Emissions
Generic disturbance rates applied to all age classes.
Carbon loss according to storage difference to the first age class.
All disturbed forests regrow and accumulate carbon immediately after disturbance approaching new equilibrium.
REMIND
General equilibrium model projecting GHG emission and transition cost of the transport, industry, building, and energy sector based on economic principles.
iv
Climate targets (1.5°C, 2.0°C).
SCM
MAGICC
Simple climate model projecting global mean temperatures driven by GHG forcings based on simplified physical principles.
%
Airborne $CO_2$ fraction determined by carbon cycle calibrated by C4MIP.
Global mean temperature

**Supplementary Figure 1. Model setup overview.** a) Model abbreviations: GCM – Global Circulation Model; DGVM – Dynamic Global Vegetation Model; IAM – Integrated Assessment Model; SCM – Simple Climate Model. b) Interchanged variables between models. Abbreviations: BioE – Bioenergy; GHG – Greenhouse Gas c) Specific models with details of their type, goal, input, and driving principles. d) Investigated uncertainty dimensions (numbered). e) Details specific to the simulation of forest dynamics and adjustments for the study's objectives. Highlighted in c) is the primary model interaction that generates the scenarios for this study. For the other models presented, previously established inputs and outputs were used.

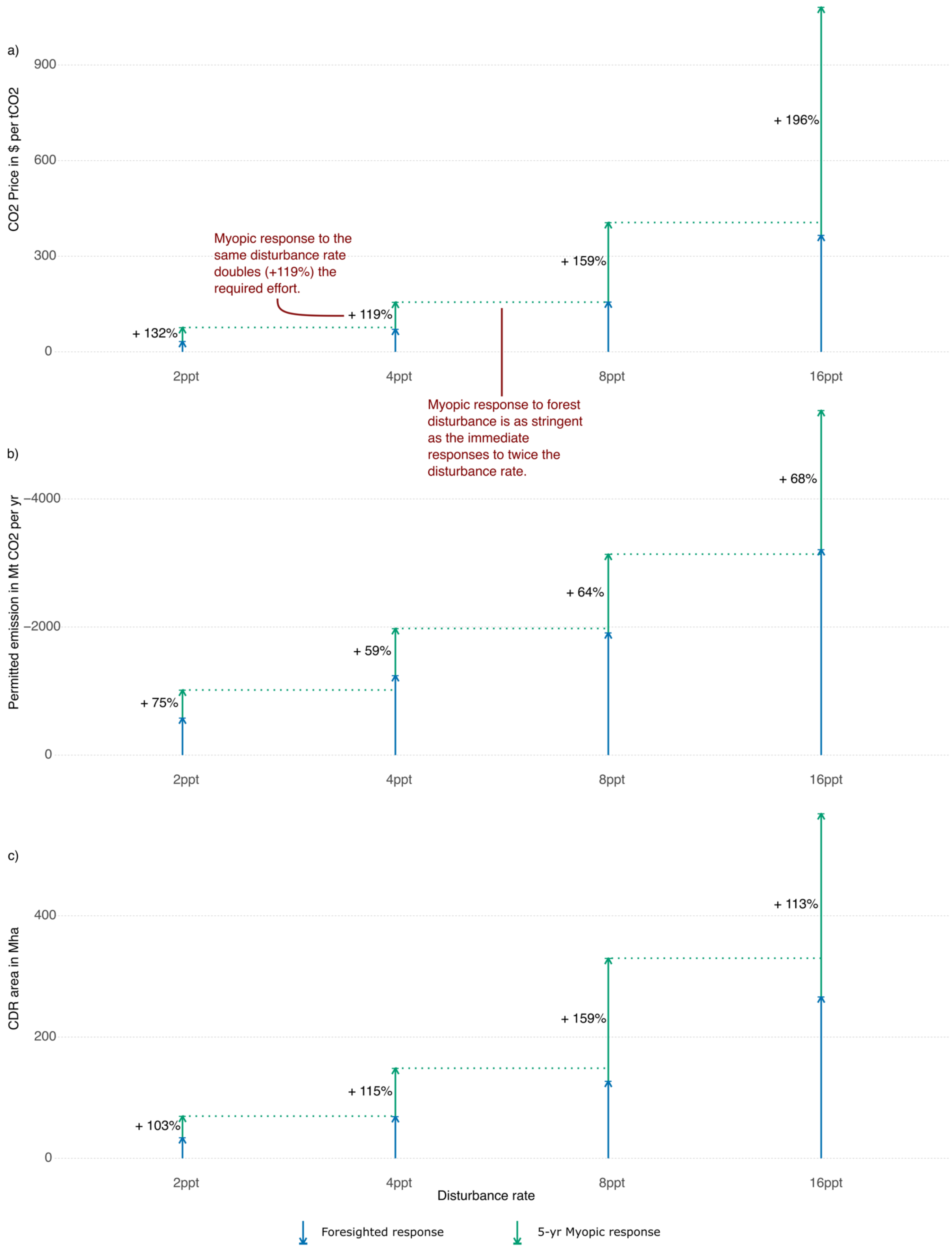
a)
CO2 Price in $ per tCO2
900
600
300
0
+ 196%
+ 159%
+ 119%
+ 132%
Myopic response to the same disturbance rate doubles (+119%) the required effort.
2ppt
4ppt
8ppt
16ppt
Myopic response to forest disturbance is as stringent as the immediate responses to twice the disturbance rate.
b)
Permitted emission in Mt CO2 per yr
−4000
−2000
0
+ 68%
+ 64%
+ 59%
+ 75%
2ppt
4ppt
8ppt
16ppt
c)
CDR area in Mha
400
200
0
+ 113%
+ 159%
+ 115%
+ 103%
2ppt
4ppt
8ppt
16ppt
Disturbance rate
Foresighted response
5-yr Myopic response

**Supplementary Figure 2. Additional mitigation response across disturbance rates.** Additional mitigation response calculated as the difference between simulations with and without disturbance (control) of three variables in 2050 to all four disturbance rates (2 to 16 parts per thousand (0.2-1.6%) per year: a) carbon price (\$/t$CO_2$); b) allowed emissions (Mt$CO_2$/year); and c) established land-based CDR area (million ha). Black arrows represent the foresighted response, calculated as the difference between simulations with and without disturbance. Turquoise arrows represent the five-year myopic response, with arrow tips indicating the response relative to the control scenario. Dotted turquoise lines connect myopic responses to the next-higher disturbance rates to aid comparison. Black numbers next to turquoise arrows indicate the percentage by which the myopic response exceeds the foresighted response (e.g., a value of +100% means the myopic response to the same disturbance rate is twice as stringent as the foresighted response). The socioeconomic setting (SSP2) and climate goal (+1.5°C) are consistent with the main manuscript.

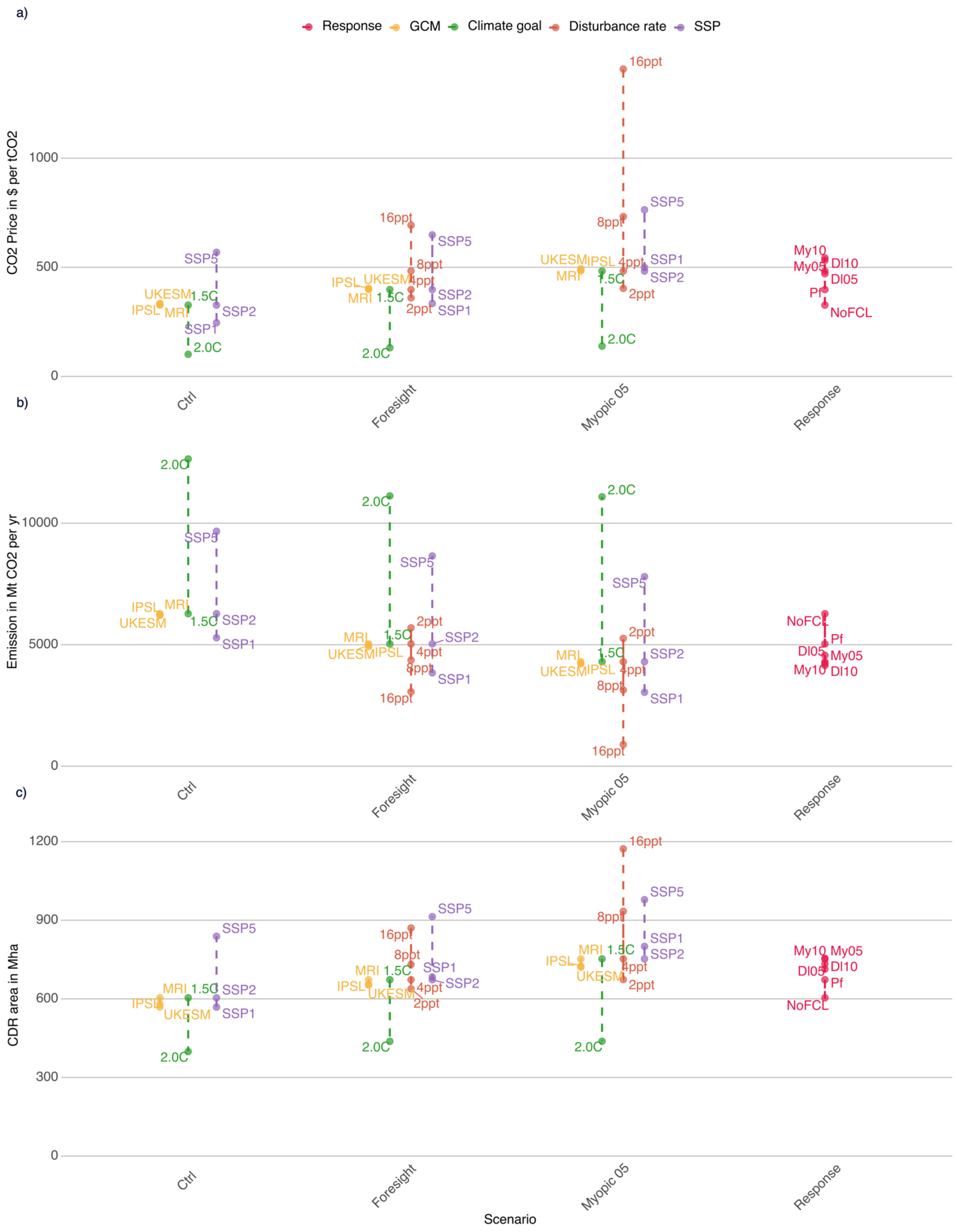

a)
Response
GCM
Climate goal
Disturbance rate
SSP
CO2 Price in $ per tCO2
1000
500
0
16ppt
8ppt
4ppt
2ppt
SSP5
SSP1
SSP2
UKESM
IPSL
MRI
1.5C
2.0C
My10
My05
Dl10
Dl05
Pf
NoFCL
Ctrl
Foresight
Myopic 05
Response
b)
Emission in Mt CO2 per yr
10000
5000
0
c)
CDR area in Mha
1200
900
600
300
0
Scenario

**Supplementary Figure 3. Uncertainty in absolute results.** Variability induced by individual uncertainty factors concerning the magnitude of three key variables in 2050. Variables explored are a) carbon price in dollar per ton $CO_2$; b) allowed emissions in $MtCO_2$ per year; and c) established land-based CDR area in million ha. From left to right the variance introduced by individual uncertainty factors is shown for the control case with no additional FCL, the foresighted response to FCL, the five-year myopic response to FCL, and all policy responses combined. Uncertainty factors (colored) are: Four policy scenarios responding to Forest Carbon Loss (FCL) plus the control case with no FCL (red); Three Global Circulation Models (GCMs, yellow); Two climate goals limiting global mean temperature to 1.5°C and 2.0°C respectively (green); Four disturbance rates inducing FCL expressed in parts per thousand (ppt) forest share disturbed per year (orange); Three Shared Socioeconomic Pathways (SSPs, purple). All uncertainty factors are explored individually with all other assumptions remaining the same. Thus, unless specified, assumptions match the ones of the main text assuming a 4ppt disturbance rate, aiming for a 1.5°C climate goal in the middle-of-the-road SSP2 scenario, using MRI GCM projections.

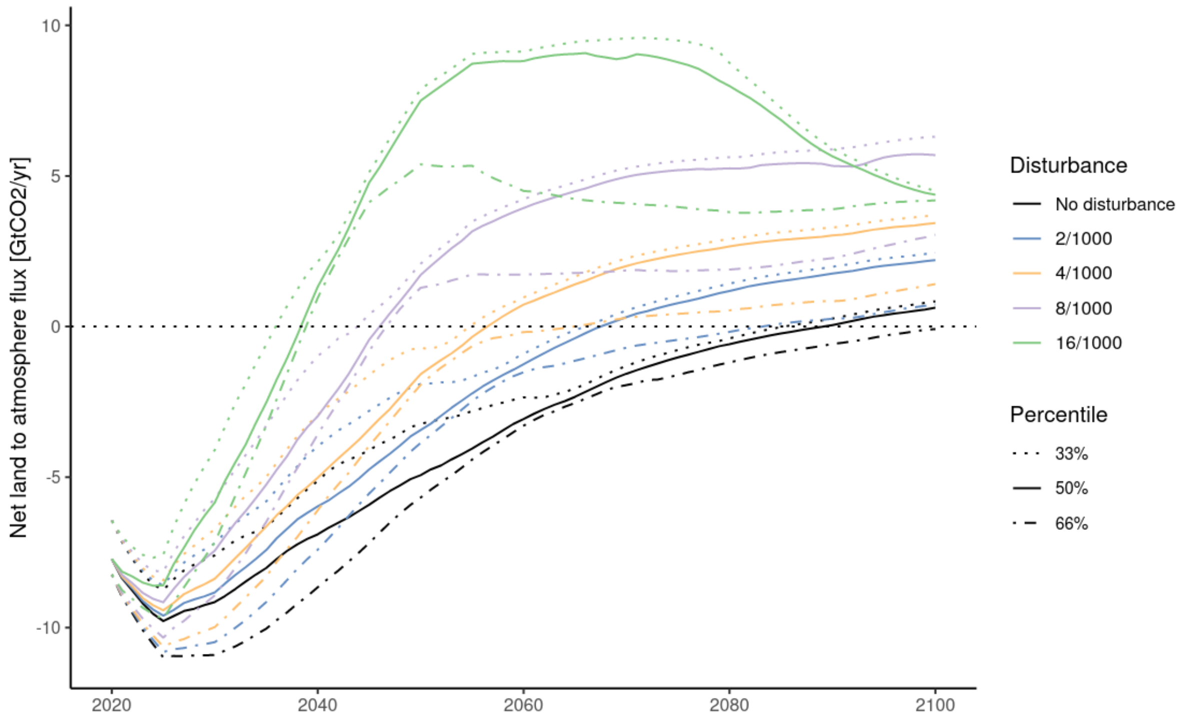


**Supplementary Figure 4. Net land sink.** Total net land to atmosphere carbon fluxes, as derived by MAGICC for four added, stylized disturbance rates: 2/1000, 4/1000, 8/1000, and 16/1000 trees per year (blue, orange, purple, green) and the control run without an additional, stylized disturbance rate (black). These fluxes include all human-induced and natural changes as well as the stylized disturbances, including the effects of changes in climate and atmospheric $CO_2$ concentration on both natural and managed land. A negative (positive) value implies that the land biosphere is a sink (source) or carbon. Solid, dashed and dash-dot lines represent respectively the 50%, 33% and 66% percentile outcomes of 600 different MAGICC parametrizations (see Methods), representing the uncertainty in the representation of both climate and carbon cycle processes in the model.

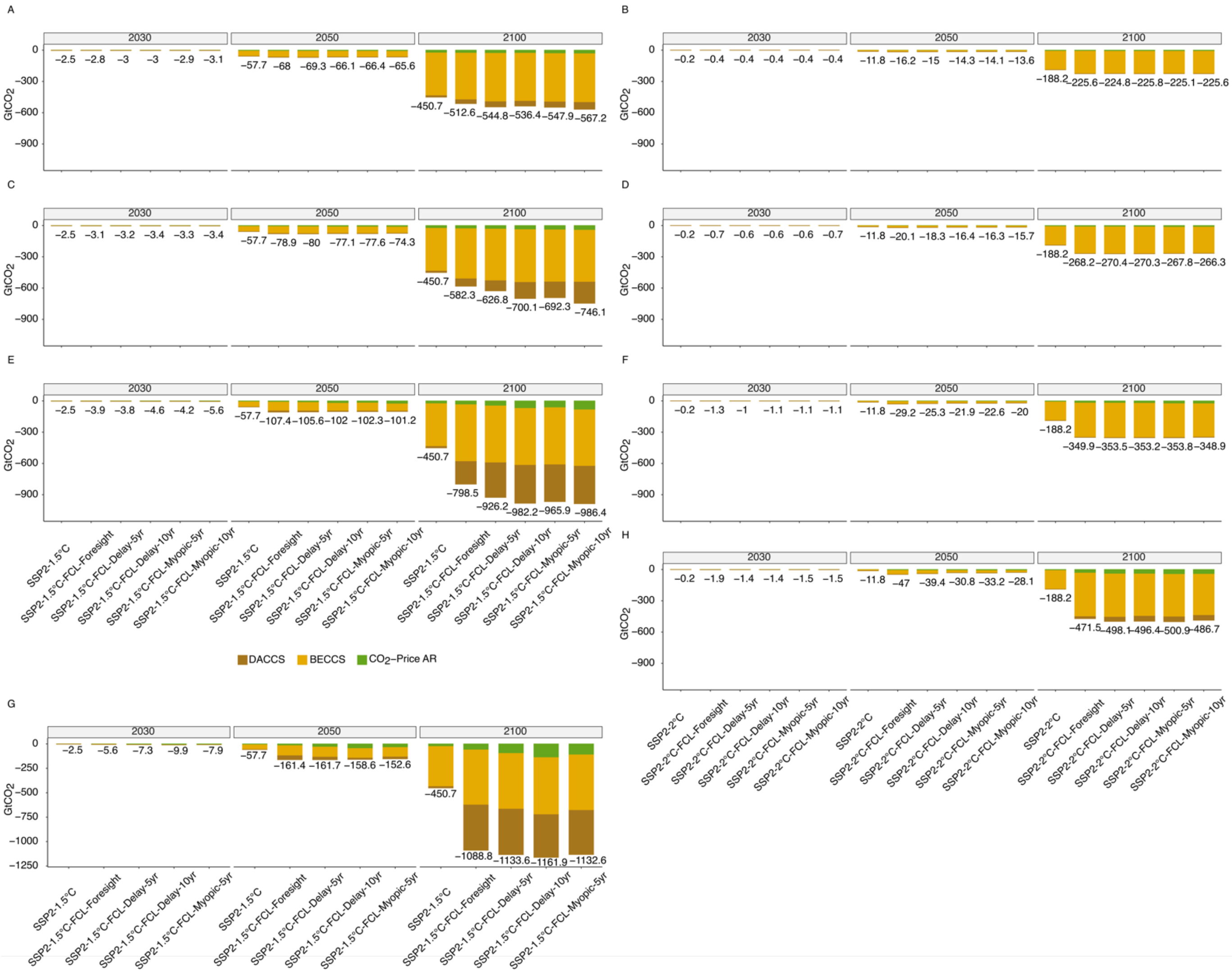

**Supplementary Figure 5. Global cumulative negative emissions.** Cumulative negative emission by source: Direct Air Carbon Capture with Storage (DACCS), Bioenergy with Carbon Capture and Storage (BECCS), and Afforestation/Reforestation (AR). The left column (sub-figures A, C, E, G) shows results of the 1.5°C mitigation scenarios. The right column (sub-figures B, D, F, H) depict results of the 2°C mitigation scenarios. Rows from top to bottom display the four disturbance rates of 2/1000 (A, B), 4/1000 (C, D), 8/1000 (E, F), and 16/1000 (G, H) trees per year. The 1.5°C mitigation scenario under 16/1000 trees per year disturbance (G) did not find a feasible solution for the "FCL-Myopic-10yr" scenario. Thus, no results are shown for the SSP2-1.5°C-FCL-Myopic-10yr scenario.

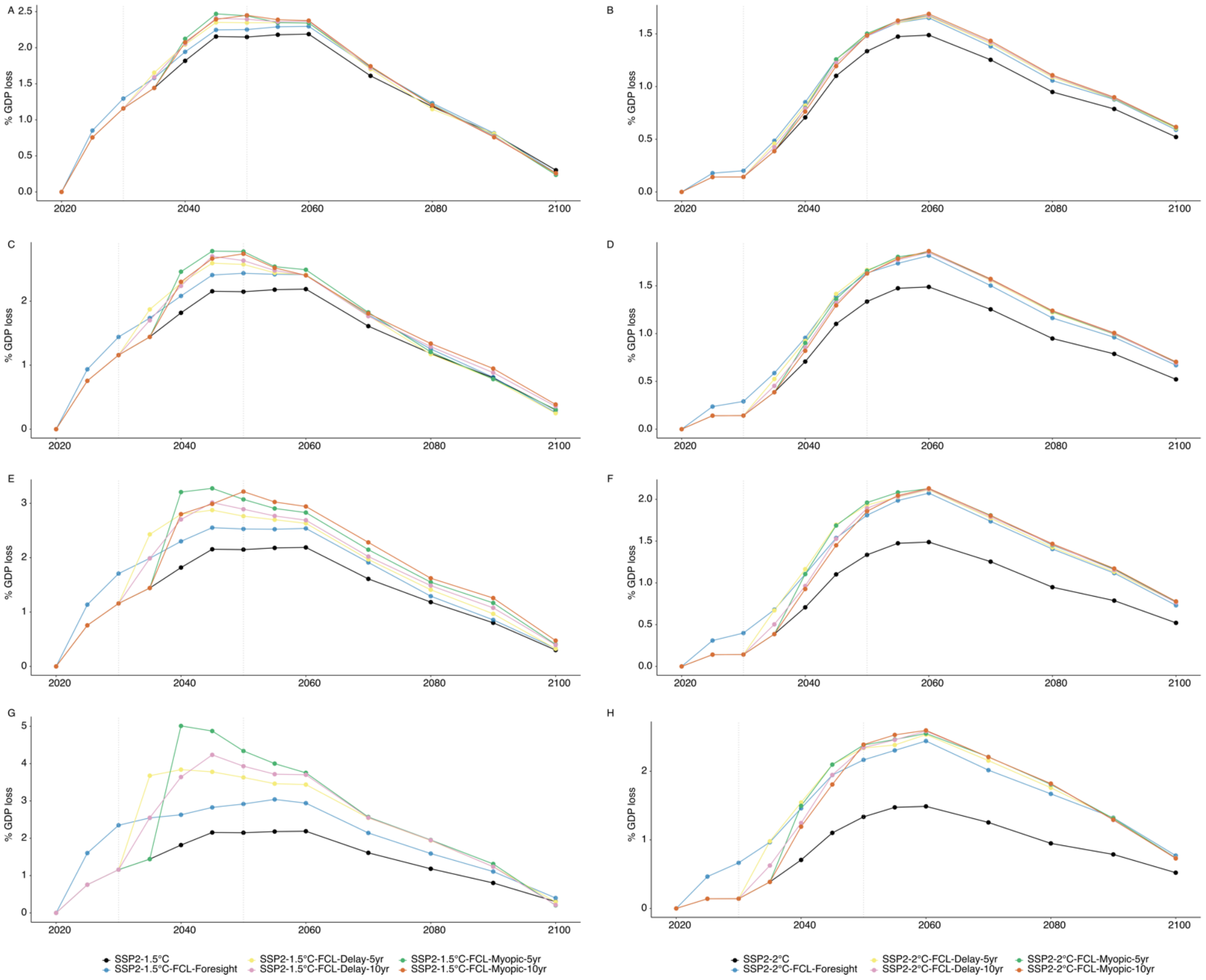


**Supplementary Figure 6. Global GDP loss development.** Percent GDP loss inferred by climate action above the baseline of currently implemented national policies (NPi). Shown are all five disturbance response policy scenarios (blue, yellow, pink, green, and orange) next to their respective control scenario (black) without forest carbon loss (FCL). The left column (sub-figures A, C, E, G) shows results of the 1.5°C mitigation scenarios. The right column (sub-figures B, D, F, H) depict results of the 2°C mitigation scenarios. Rows from top to bottom display the four disturbance rates of 2/1000 (A, B), 4/1000 (C, D), 8/1000 (E, F), and 16/1000 (G, H) trees per year. The 1.5°C mitigation scenario under 16/1000 trees per year disturbance (G) did not find a feasible solution for the "FCL-Myopic-10yr" scenario. Thus, no results are shown for the SSP2-1.5°C-FCL-Myopic-10yr scenario.

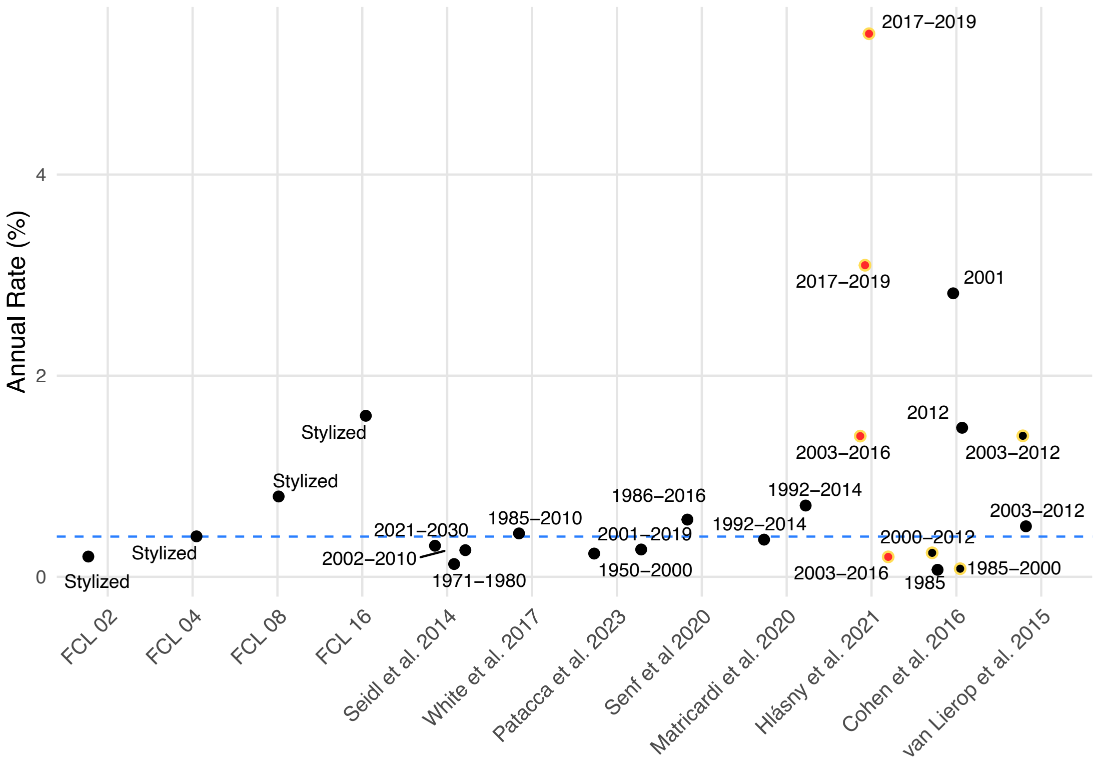


| Source | Annual rate [%] | Region | Disturbance factors | Period | Regional limitation | Factor limitation |
|---|---|---|---|---|---|---|
| FCL 02 | 0.2 | Global | Indirect + direct | Stylized future assumption | | |
| FCL 04 | 0.4 | Global | Indirect + direct | Stylized future assumption | | |
| FCL 08 | 0.8 | Global | Indirect + direct | Stylized future assumption | | |
| FCL 16 | 1.6 | Global | Indirect + direct | Stylized future assumption | | |
| Seidl et al. 2014 | 0.126 | Europe only | Indirect only | 1971-1980 | | |
| Seidl et al. 2014 | 0.264 | Europe only | Indirect only | 2002-2010 | | |
| Seidl et al. 2014 | 0.311 | Europe only | Indirect only | 2021-2030 | | |
| White et al. 2017 | 0.43 | Canada only | Wildfire and harvest only | 1985-2010 | | |
| Patacca et al. 2023 | 0.23 | Europe only | Indirect only | 1950-2000 | | |
| Patacca et al. 2023 | 0.27 | Europe only | Indirect only | 2001-2019 | | |
| Senf et al 2020 | 0.57 | Europe only | Indirect + direct | 1986-2016 | | |
| Matricardi et al. 2020 | 0.37 | Brazilian Amazon only | Degradation as sideeffect of direct | 1992-2014 | | |
| Matricardi et al. 2020 | 0.71 | Brazilian Amazon only | Combined degradation and direct | 1992-2014 | | |
| Hlásny et al. 2021 | 0.2 | Czech Republic only | Bark Beetle only | 2003-2016 | *Limited region! | ^Single factor! |
| Hlásny et al. 2021 | 1.4 | Czech Republic only | Bark Beetle only | 2003-2016 | *Limited region! | ^Single factor! |
| Hlásny et al. 2021 | 3.1 | Czech Republic only | Bark Beetle only | 2017-2019 | *Limited region! | ^Single factor! |
| Hlásny et al. 2021 | 5.4 | Czech Republic only | Bark Beetle only | 2017-2020 | *Limited region! | ^Single factor! |
| Cohen et al. 2016 | 0.07 | USA only | Decline only | 1985 | | |
| Cohen et al. 2016 | 1.48 | USA only | Decline only | 2012 | | |
| Cohen et al. 2016 | 2.82 | USA only | Decline only | 2001 | | |
| Cohen et al. 2016 | 0.08 | USA only | Fire only | 1985-2000 | | ^Single factor! |
| Cohen et al. 2016 | 0.24 | USA only | Fire only | 2000-2012 | | ^Single factor! |
| van Lierop et al. 2015 | 1.4 | Global | Fire only | 2003-2012 | | ^Single factor! |
| van Lierop et al. 2015 | 0.5 | Global | Pest, disease, severe weather | 2003-2012 | | |

*Limited region! Likely not applicable to other biomes / regions. Overestimation more likely.
^Single factor! Additional sources could be additive. Underestimation more likely.

**Supplementary Figure 7. Disturbance rate comparison.** (Top) Annual forest disturbance rates of the four stylized future assumptions of this study (FCL02 to FCL16) and 20 estimates of eight studies[1–8]. Red-filled dots denote literature estimates done on a limited geographical scope. Thus, their estimates might likely not be applicable to other biomes or regions making it more likely to be an overestimation of the global disturbance rate. Yellow-lined dots denote literature estimates done for only one single disturbance factor. Thus, additional factors might be additive to this single factor making an underestimation of the overall disturbance rate more likely. The blue dashed line marks the 0.4% (FCL04) disturbance rate highlighted in the main text. The table (bottom) provides additional information on the literature sources, their specific annual disturbance rate, region, included disturbance factors, assessed period, and their regional & factor limitations.

**Supplementary Table 1. Interest rate evolution.** Interest rate evolution in percent per year between 2020 and 2100 of SSP1, SSP2, and SSP5 of the 12 world regions of REMIND-MAgPIE. Region abbreviations: Canada, Australia and New Zealand: CAZ; China: CHA; European Union: EUR; India: IND; Japan: JPN; Latin America: LAM; Middle East and North Africa: MEA; non-EU member states: NEU; other Asia: OAS; reforming countries: REF; sub-Saharan Africa: SSA; United States: USA).

**SSP1**

| | Region | | | | | | | | | | | |
|---|---|---|---|---|---|---|---|---|---|---|---|---|
| Year | CAZ | CHA | EUR | IND | JPN | LAM | MEA | NEU | OAS | REF | SSA | USA |
| y2020 | 4.00 | 4.00 | 4.00 | 7.44 | 4.00 | 4.62 | 5.58 | 4.08 | 6.88 | 5.05 | 8.96 | 4.00 |
| y2025 | 4.00 | 4.00 | 4.00 | 6.64 | 4.00 | 4.53 | 5.41 | 4.02 | 6.43 | 4.83 | 8.93 | 4.00 |
| y2030 | 4.00 | 4.00 | 4.00 | 5.49 | 4.00 | 4.35 | 4.95 | 4.00 | 6.03 | 4.42 | 8.51 | 4.00 |
| y2035 | 4.00 | 4.00 | 4.00 | 4.00 | 4.00 | 4.19 | 4.63 | 4.00 | 5.52 | 4.18 | 7.91 | 4.00 |
| y2040 | 4.00 | 4.00 | 4.00 | 4.00 | 4.00 | 4.09 | 4.43 | 4.00 | 5.07 | 4.09 | 7.10 | 4.00 |
| y2045 | 4.00 | 4.00 | 4.00 | 4.00 | 4.00 | 4.03 | 4.26 | 4.00 | 4.59 | 4.02 | 6.12 | 4.00 |
| y2050 | 4.00 | 4.00 | 4.00 | 4.00 | 4.00 | 4.01 | 4.15 | 4.00 | 4.28 | 4.00 | 5.22 | 4.00 |
| y2055 | 4.00 | 4.00 | 4.00 | 4.00 | 4.00 | 4.00 | 4.09 | 4.00 | 4.17 | 4.00 | 4.46 | 4.00 |
| y2060 | 4.00 | 4.00 | 4.00 | 4.00 | 4.00 | 4.00 | 4.03 | 4.00 | 4.10 | 4.00 | 4.16 | 4.00 |
| y2065 | 4.00 | 4.00 | 4.00 | 4.00 | 4.00 | 4.00 | 4.01 | 4.00 | 4.10 | 4.00 | 4.12 | 4.00 |
| y2070 | 4.00 | 4.00 | 4.00 | 4.00 | 4.00 | 4.00 | 4.01 | 4.00 | 4.09 | 4.00 | 4.11 | 4.00 |
| y2075 | 4.00 | 4.00 | 4.00 | 4.00 | 4.00 | 4.00 | 4.01 | 4.00 | 4.09 | 4.00 | 4.11 | 4.00 |
| y2080 | 4.00 | 4.00 | 4.00 | 4.00 | 4.00 | 4.00 | 4.01 | 4.00 | 4.09 | 4.00 | 4.10 | 4.00 |
| y2085 | 4.00 | 4.00 | 4.00 | 4.00 | 4.00 | 4.00 | 4.01 | 4.00 | 4.09 | 4.00 | 4.09 | 4.00 |
| y2090 | 4.00 | 4.00 | 4.00 | 4.00 | 4.00 | 4.00 | 4.01 | 4.00 | 4.09 | 4.00 | 4.08 | 4.00 |
| y2095 | 4.00 | 4.00 | 4.00 | 4.00 | 4.00 | 4.00 | 4.01 | 4.00 | 4.09 | 4.00 | 4.08 | 4.00 |
| y2100 | 4.00 | 4.00 | 4.00 | 4.00 | 4.00 | 4.00 | 4.01 | 4.00 | 4.09 | 4.00 | 4.07 | 4.00 |

**SSP2**

| | Region | | | | | | | | | | | |
|---|---|---|---|---|---|---|---|---|---|---|---|---|
| Year | CAZ | CHA | EUR | IND | JPN | LAM | MEA | NEU | OAS | REF | SSA | USA |
| y2020 | 4.00 | 4.00 | 4.00 | 7.44 | 4.00 | 4.62 | 5.58 | 4.08 | 6.88 | 5.05 | 8.96 | 4.00 |
| y2025 | 4.00 | 4.00 | 4.00 | 6.64 | 4.00 | 4.53 | 5.41 | 4.02 | 6.43 | 4.83 | 8.93 | 4.00 |
| y2030 | 4.00 | 4.00 | 4.00 | 5.80 | 4.00 | 4.41 | 5.05 | 4.00 | 6.16 | 4.54 | 8.68 | 4.00 |
| y2035 | 4.00 | 4.00 | 4.00 | 4.97 | 4.00 | 4.29 | 4.82 | 4.00 | 5.92 | 4.31 | 8.37 | 4.00 |
| y2040 | 4.00 | 4.00 | 4.00 | 4.04 | 4.00 | 4.20 | 4.65 | 4.00 | 5.63 | 4.19 | 7.99 | 4.00 |
| y2045 | 4.00 | 4.00 | 4.00 | 4.00 | 4.00 | 4.11 | 4.51 | 4.00 | 5.34 | 4.13 | 7.50 | 4.00 |
| y2050 | 4.00 | 4.00 | 4.00 | 4.00 | 4.00 | 4.06 | 4.41 | 4.00 | 5.06 | 4.06 | 6.91 | 4.00 |
| y2055 | 4.00 | 4.00 | 4.00 | 4.00 | 4.00 | 4.03 | 4.26 | 4.00 | 4.71 | 4.00 | 6.19 | 4.00 |
| y2060 | 4.00 | 4.00 | 4.00 | 4.00 | 4.00 | 4.01 | 4.16 | 4.00 | 4.41 | 4.00 | 5.58 | 4.00 |
| y2065 | 4.00 | 4.00 | 4.00 | 4.00 | 4.00 | 4.00 | 4.10 | 4.00 | 4.34 | 4.00 | 4.95 | 4.00 |
| y2070 | 4.00 | 4.00 | 4.00 | 4.00 | 4.00 | 4.00 | 4.03 | 4.00 | 4.24 | 4.00 | 4.46 | 4.00 |
| y2075 | 4.00 | 4.00 | 4.00 | 4.00 | 4.00 | 4.00 | 4.01 | 4.00 | 4.14 | 4.00 | 4.20 | 4.00 |
| y2080 | 4.00 | 4.00 | 4.00 | 4.00 | 4.00 | 4.00 | 4.01 | 4.00 | 4.08 | 4.00 | 4.15 | 4.00 |
| y2085 | 4.00 | 4.00 | 4.00 | 4.00 | 4.00 | 4.00 | 4.01 | 4.00 | 4.08 | 4.00 | 4.14 | 4.00 |
| y2090 | 4.00 | 4.00 | 4.00 | 4.00 | 4.00 | 4.00 | 4.01 | 4.00 | 4.08 | 4.00 | 4.14 | 4.00 |
| y2095 | 4.00 | 4.00 | 4.00 | 4.00 | 4.00 | 4.00 | 4.01 | 4.00 | 4.08 | 4.00 | 4.14 | 4.00 |
| y2100 | 4.00 | 4.00 | 4.00 | 4.00 | 4.00 | 4.00 | 4.01 | 4.00 | 4.08 | 4.00 | 4.13 | 4.00 |

**SSP5**

| | Region | | | | | | | | | | | |
|---|---|---|---|---|---|---|---|---|---|---|---|---|
| Year | CAZ | CHA | EUR | IND | JPN | LAM | MEA | NEU | OAS | REF | SSA | USA |
| y2020 | 4.00 | 4.00 | 4.00 | 7.44 | 4.00 | 4.62 | 5.58 | 4.08 | 6.88 | 5.05 | 8.96 | 4.00 |
| y2025 | 4.00 | 4.00 | 4.00 | 6.64 | 4.00 | 4.53 | 5.41 | 4.02 | 6.43 | 4.83 | 8.93 | 4.00 |
| y2030 | 4.00 | 4.00 | 4.00 | 5.27 | 4.00 | 4.32 | 4.89 | 4.00 | 5.94 | 4.36 | 8.42 | 4.00 |
| y2035 | 4.00 | 4.00 | 4.00 | 4.00 | 4.00 | 4.13 | 4.53 | 4.00 | 5.30 | 4.14 | 7.58 | 4.00 |
| y2040 | 4.00 | 4.00 | 4.00 | 4.00 | 4.00 | 4.05 | 4.33 | 4.00 | 4.72 | 4.03 | 6.47 | 4.00 |
| y2045 | 4.00 | 4.00 | 4.00 | 4.00 | 4.00 | 4.01 | 4.17 | 4.00 | 4.28 | 4.00 | 5.35 | 4.00 |
| y2050 | 4.00 | 4.00 | 4.00 | 4.00 | 4.00 | 4.00 | 4.10 | 4.00 | 4.17 | 4.00 | 4.46 | 4.00 |
| y2055 | 4.00 | 4.00 | 4.00 | 4.00 | 4.00 | 4.00 | 4.01 | 4.00 | 4.10 | 4.00 | 4.16 | 4.00 |
| y2060 | 4.00 | 4.00 | 4.00 | 4.00 | 4.00 | 4.00 | 4.01 | 4.00 | 4.10 | 4.00 | 4.12 | 4.00 |
| y2065 | 4.00 | 4.00 | 4.00 | 4.00 | 4.00 | 4.00 | 4.01 | 4.00 | 4.10 | 4.00 | 4.11 | 4.00 |
| y2070 | 4.00 | 4.00 | 4.00 | 4.00 | 4.00 | 4.00 | 4.01 | 4.00 | 4.10 | 4.00 | 4.10 | 4.00 |
| y2075 | 4.00 | 4.00 | 4.00 | 4.00 | 4.00 | 4.00 | 4.01 | 4.00 | 4.10 | 4.00 | 4.09 | 4.00 |
| y2080 | 4.00 | 4.00 | 4.00 | 4.00 | 4.00 | 4.00 | 4.01 | 4.00 | 4.10 | 4.00 | 4.08 | 4.00 |
| y2085 | 4.00 | 4.00 | 4.00 | 4.00 | 4.00 | 4.00 | 4.01 | 4.00 | 4.10 | 4.00 | 4.07 | 4.00 |
| y2090 | 4.00 | 4.00 | 4.00 | 4.00 | 4.00 | 4.00 | 4.01 | 4.00 | 4.09 | 4.00 | 4.08 | 4.00 |
| y2095 | 4.00 | 4.00 | 4.00 | 4.00 | 4.00 | 4.00 | 4.01 | 4.00 | 4.09 | 4.00 | 4.08 | 4.00 |
| y2100 | 4.00 | 4.00 | 4.00 | 4.00 | 4.00 | 4.00 | 4.01 | 4.00 | 4.09 | 4.00 | 4.08 | 4.00 |

**Supplementary References**


1. Seidl, R., Schelhaas, M. J., Rammer, W. & Verkerk, P. J. Increasing forest disturbances in Europe and their impact on carbon storage. *Nat Clim Chang* 4, 806–810 (2014).
2. White, J. C., Wulder, M. A., Hermosilla, T., Coops, N. C. & Hobart, G. W. A nationwide annual characterization of 25 years of forest disturbance and recovery for Canada using Landsat time series. *Remote Sens Environ* 194, 303–321 (2017).
3. Patacca, M. *et al.* Significant increase in natural disturbance impacts on European forests since 1950. *Glob Chang Biol* 29, 1359–1376 (2023).
4. Senf, C. & Seidl, R. Mapping the forest disturbance regimes of Europe. *Nat Sustain* 4, 63–70 (2020).
5. Matricardi, E. A. T. *et al.* Long-term forest degradation surpasses deforestation in the Brazilian Amazon. *Science (1979)* 369, 1378–1382 (2020).
6. Hlásny, T. *et al.* Devastating outbreak of bark beetles in the Czech Republic: Drivers, impacts, and management implications. *For Ecol Manage* 490, 119075 (2021).
7. Cohen, W. B. *et al.* Forest disturbance across the conterminous United States from 1985–2012: The emerging dominance of forest decline. *For Ecol Manage* 360, 242–252 (2016).
8. van Lierop, P., Lindquist, E., Sathyapala, S. & Franceschini, G. Global forest area disturbance from fire, insect pests, diseases and severe weather events. *For Ecol Manage* 352, 78–88 (2015).
